# Investigation of Bioactive Components in New Resistant Hungarian Tomato Hybrids

**DOI:** 10.3390/plants11233408

**Published:** 2022-12-06

**Authors:** Barbara Schmidt-Szantner, Mária Berki, Éva Lengyel-Kónya, Péter Milotay, Ágnes Molnár-Mondovics, Hussein G. Daood, Rita Tömösközi-Farkas

**Affiliations:** 1 Vegetable Research Center, Hungarian University of Agriculture and Life Sciences, 6000 Kecskemét, Hungary; 2 Food Science Research Group, Food Science and Technology Institute, Hungarian University of Agriculture and Life Sciences, 1118 Budapest, Hungary; 3 Szent Isván Campus, Hungarian University of Agriculture and Life Sciences, 2100 Gödöllő, Hungary

**Keywords:** tomato, bioactive metabolites, resistance, growing technology

## Abstract

The aim of the present work was to evaluate the influence of genetic impact on the content of some characteristic secondary metabolites in tomato fruits. The study was conducted to screen 14 different tomato genotypes for antioxidant capacity and quality attributes (polyphenols, tocopherols, carotenoids, vitamin C) to select the genetic lines with high biological value, multiple disease resistance, and high yield capacity for further usage in crop improvement programs. Significant differences were found among the different hybrids and cultivation seasons for the phytochemical content of the fruits. The varieties developed by breeding to increase their resistance were found to differ significantly. During a 3-year cultivation experiment, the level of lycopene in freshly harvested fruits ranged between 10.38 and 63.23 mg.kg^−1^ fwt for outdoor-cultivated Naik114 and Naik352, respectively. As for β-carotene, its content varied between 0.49 mg.kg^−1^ for Unorosso and 11.66 mg.kg^−1^ for Cherrola F1. The highest level of vitamin C (702.59 mg.kg^−1^) was recorded for Cherrola F1, while the lowest level (1.36.86 mg.kg^−1^) was determined in fruits of Unorosso. Neither polyphenol nor vitamin C showed positive correlation with antioxidant activity. In the three cultivation seasons, the highest concentration of polyphenols (579.19–804.12 mg.kg^−1^ fwt) was determined in fruits of outdoor-cultivated Cherolla F1 variety. The α- and γ-tocopherol content varied between 2.56 and 18.99 mg.kg^−1^, and 0.28 and 9.35 mg.kg^−1^, respectively, in fresh tomato fruit. Among the newly bred resistant varieties, the ZKI114 variety was proved to have outstanding features concerning the amounts of the bioactive components.

## 1. Introduction

Owing to their considerably high content of phytochemicals and other bioactive ingredients, vegetables and fruits are important in human nutrition. Their health protecting effects are due to the presence of functional components, e.g., antioxidative compounds, such as polyphenols, carotenoids, tocopherols, vitamins, certain peptides, saccharides, etc. [1,2,3]. The World Health Organization (WHO and the Food and Agriculture Organization of the United Nations (FAO) have emphasized the role of fruits and vegetables, with special regard to the biologically active substances of plant origin, in the prevention of different non-communicable diseases and cancer [4]. In this respect, tomato (*Solanum lycopersicum* L.) is an important source of health-promoting compounds that can reduce the development of cardiovascular disease, gastrointestinal tumors, inflammatory processes, and hypertension. These properties have been associated with the presence of hydrophilic (ascorbic acid and polyphenols) [5] and lipophilic antioxidants, including carotenoids (mainly lycopene and β-carotene) and vitamin E [6]. These functional components can regulate the mechanism of detoxification [7] as well. Carotenoids possess an apoptotic-inducing effect in cancer cells [8], and lycopene has been demonstrated to reduce oxidized-LDL cholesterol levels [9].

Epidemiological studies have highlighted the potential beneficial role of vitamin E intake on inflammation-related diseases, cardiac functionality, reduction in risk of Alzheimer’s disease, type 2 diabetes, prostate cancer, and prevention of retinopathy and cataracts. It has been proved that tocopherols from tomato consumption can have a different effect on health than vitamin E supplementation, because other antioxidant components are also present in tomatoes [10].

It has been proved that the concentration of the health-promoting bioactive compounds and the antioxidant activity of tomato extracts are strongly influenced by several biotic and abiotic factors, such as the genotype, ripening stage of the fruit, farming technology, and climate conditions [11,12,13].

Induced resistance against plant viruses has been studied for many years [14]. Induced resistance has advantages for combating viruses, because no agrochemicals analogous to fungicides or insecticides exist that can be used to prevent virus diseases under field conditions. Several new components have been identified in resistant tomato cultivars that may be responsible for the development of resistance, such as methylated forms of flavonoid myricetin [15], or α-tomatine [16]. Wojciechowska and coworkers [17] have shown clear differences in the metabolic profiles that were paralleled by differences in resistance toward Alternaria colonization. One of the genotypes was more resistant against A. alternata infection and contained high amounts of chlorogenic acid, in contrast to the other genotype which was sensitive against infection.

Our main objective was to characterize professional hybrids, test hybrids, and parental lines (14 genotypes), with special emphasis on some metabolites with health-promoting effects (lycopene, β-carotene, α-tocopherols, γ-tocopherols, total polyphenol content, DPPH scavenging activity, vitamin C). The main aspects of the experiments and the various measurements were to compare and find relationships among all the data and the new cultivars, which varied in fruit size (small (3), medium (8), large (3)), type (industrial (9), fresh market (5)), resistance (multiresistance (9), susceptible (5)) and agrotechnology (open field, greenhouse).

## 2. Results

### 2.1. Content of Carotenoids

The characteristic red color of tomato is a result of combined carotenoid pigments, of which lycopene is the most important. The tomato color is determined by the pigmentation of the skin and flesh [18]. Regulation of carotenoid biosynthesis and the accumulation of lycopene during tomato fruit ripening are widely studied [19,20]. Saini et al. [13] recorded an increase in carotenoids and lycopene contents during ripening.

The human diet contains two sources for vitamin A: preformed vitamin A (retinol and retinyl esters) and provitamin A carotenoids, which are plant pigments that the body converts in the liver into vitamin A. The main provitamin A carotenoids in the human diet are β-carotene, α-carotene, and β-cryptoxanthin. Other carotenoids in food, such as lycopene, lutein, and zeaxanthin, are not converted into vitamin A and are referred to as non-provitamin A carotenoids; they may have other important activities not involving vitamin A formation [21,22].

Recommended dietary allowance (RDA) for vitamin A is given as retinol activity equivalents (RAE) to account for the different bioactivities of retinol and provitamin A carotenoids, all of which are converted by the body into retinol [23]. One ug RAE is equivalent to 1 ug retinol, 2 ug supplemental β-carotene, 12 ug dietary β-carotene, or 24 ug dietary α-carotene or β-cryptoxanthin. The RDA for vitamin A is 7–900 ug RAE for healthy adults and 3–900 RAE for children, depending on age [23].

Concerning human nutrition and tomato quality, β-carotene and lycopene are of special interest. The β-carotene content of the examined varieties varied between 0.65 and 11.6 mg.kg^−1^. This level is in the range 5–12 mg.kg^−1^ reported in the relevant literature [24,25,26,27]. The highest β-carotene value was measured in the Cherrola F1 variety in 2015 (11.6 mg.kg^−1^), while the lowest value was measured in the Mokka F1 variety in 2016 (0.65 mg.kg^−1^). Based on the average of the three years, Cherrola F1 was also the richest one in β-carotene (6.03 mg.kg^−1^), while the NAIK114 big-fruited fresh market line contained the least (1.91 mg.kg^−1^).

There is currently no recommended daily intake for lycopene [28]. However, from a study by Chen et al. [29], intakes between 8 and 21 mg per day appear to be most beneficial. Most red and pink foods contain some lycopene, but tomatoes and tomato-based foods are the richest sources of this nutrient. According to Helyes et al. [30] the lycopene content of 16 different tomato varieties in Hungary ranged between 39.3 and 171.0 mg.kg^−1^. During our 3-year experiments, the measured lycopene values ranged from 10.38 to 63.23 mg kg^−1^, which correspond to the literature values. Summarizing results from both the field and plastic house samples, the processing hybrid NAIK3992 proved to be the richest in lycopene; the 3-year average lycopene level was 47.02 mg.kg^−1^, while the NAIK114 variety had the least amount, on average 16.09 mg.kg^−1^. In the cherry group, NAIK 3254 F1 was best for lycopene and carotene content.

Significant differences were found in lycopene concentration between the years for both outdoor and indoor cultivation. The lowest average values were measured in 2014 (29.07 mg.kg^−1^ lycopene, 3.05 mg.kg^−1^ β-carotene), while in the second and third year the average amount of carotenoids was significantly higher compared to 2014 (39.32 mg.kg^−1^, 3.99 mg.kg^−1^ in 2015 and 37.05 mg.kg^−1^, 2.07 mg.kg^−1^ in 2016 for lycopene and β-carotene, respectively) (Table 1). These results affirmed the high impact of seasonal (climate) variations on the content of bioactive carotenoids in tomatoes. The climate factors most likely to affect carotenoid content and composition in tomato may include light intensity, temperature, and water supply, particularly at a few weeks before harvest [31].

Concerning the effect of cultivation method on carotenoid content in tomatoes, our measurements in 2015 and in 2016 did not show a significant difference between the two cultivation methods in lycopene content (2015—PH: 41.47 ± 76.78 mg.kg^−1^, OF: 36.63 ± 9.65 mg.kg^−1^, 2016—PH: 43.52 ± 7.1 mg.kg^−1^, OF:26.71 ± 6.75 mg.kg^−1^ mean values), however, the average amount of β-carotene in 2015 was higher in outdoor cultivation samples (PH: 3.58 ± 1.35 mg.kg^−1^, OF: 5.06 ± 2.72 mg.kg^−1^ mean values), but this difference was not significant (Table 1). These results are not consistent with those of some other studies. Brandt and coworkers [32] observed significantly higher lycopene content in tomato harvested in a greenhouse (83.0 mg.kg^−1^) than in an open field (59.2 mg.kg^−1^) at every harvesting time. Keyhaninejad and coworkers [33] investigated the relationship between light levels in the growth environment and the carotenoid levels that accumulated in mature fruit and leaves. The foliar carotenoid increased approximately twofold with increased light, whereas carotenoid content in fruit decreased two- to threefold with increased light. All cultivars showed identical trends with light despite having cultivar-specific carotenoid accumulation patterns in their fruit. Carotenoid content of fruits is higher in greenhouse samples based on data from the literature.

When industrial varieties are compared with fresh market ones, the average lycopene content of processing and cherry varieties was found to be higher (industrial: 39.96 ± 3.69 mg kg^−1^ mean lycopene value, fresh market: 28.18 ± 6.3 mg.kg^−1^), but no difference was found in the β-carotene content (industrial: 3.15 ± 0.3 mg.kg^−1^, fresh market: 3.88 ± 0.8 mg.kg^−1^).

It is interesting that no correlation was found between the lycopene content and the fruit size; moreover, there was no significant difference between resistant and susceptible tomato varieties in the lycopene content.

### 2.2. Content of Tocopherols

The vitamin E group consists of four tocopherols (α, β, δ, and γ), and four tocotrienols (α, β, δ, and γ) that are non-enzymatic lipid-soluble antioxidants. In both food and human tissues, α-tocopherol and γ-tocopherol are the more characteristic forms [34,35], so in our studies we focused on these two tocopherol components. Some research indicated a greater antioxidant potential of tocotrienols than of tocopherols; however, compared to tocopherols, they are less orally bioavailable, and they are more reactive and can form potentially cytotoxic adducts [36]. The RDA for vitamin E for males and females ages 14 years and older is 15 mg daily (or 22 international units, IU), including pregnant. Lactating women need slightly more at 19 mg (28 IU) daily [37].

Although tocopherol concentration is relatively low in tomatoes, the high daily consumption of fresh and processed products with high dry matter content can increase the daily intake to achieve or to be close to the recommended dietary intake (RDI) of the biologically active form of vitamin E. This aspect is to be considered in breeding programs for industrial tomato varieties [38]. The amounts of tocopherols in the tomato fruit depends significantly on many biotic and abiotic factors. Based on the results of Raiola and coworkers [34], irrigation, light, and NaCl levels have a great effect on the biosynthesis of tocopherols in tomato. The level of α-tocopherol increased during ripening in all tissues, though its increase was largest between light red to red stages. Based on the data found in the literature, the amount of α-tocopherol in tomatoes varies between 2 and 14.7 mg.kg^−1^ [39,40,41,42], which agrees with the values found in the examined varieties. During the 3-year study, the α-tocopherol content varied between 2.56 and 18.99 mg.kg^−1^ fwt, while γ-tocopherol content varied between 0.28 and 9.35 mg.kg^−1^ in fresh tomato fruit. The variety NAIK3992 contained the highest amount of all tocopherols (26.6 mg.kg^−1^ fwt) in 2014, the variety NAIK 3254 in 2015 (15.01 mg.kg^−1^ fwt), and the variety Aragon F1 in 2016 (12.42 mg.kg^−1^ fwt) (Table 2).

In the open field experiments during the first year (2014), results showed the highest content of tocopherol derivatives, α- and γ-tocopherol (14.49 mg.kg^−1^, 4.98 mg.kg^−1^ mean values of 14 cultivars, respectively) in the samples. The lowest values were determined in the third year (7.55 mg.kg^−1^, 1.17 mg.kg^−1^, as mean values, respectively) in almost all varieties (Table 2). Differences between seasons proved to be significant for most varieties.

The effect of indoor conditions and open cultivation models were compared on the antioxidant activity of cherry and ordinary tomatoes [43], and the results showed that the antioxidant content, such as carotenoids and tocopherols, and the antioxidant activity of tomatoes were affected not only by the changes in environmental factors, but also by the varieties. Our measurements proved that tomatoes from outdoor cultivation contained significantly higher α-tocopherol content than that of the indoor-grown varieties (Table 2). We determined higher average concentration of α-tocopherol in 2015 (PH: 8.30 ± 1.62 mg.kg^−1^, OF: 9.57 ± 1.93 mg.kg^−1^) and 2016 (PH: 5.09 ± 1.67 mg.kg^−1^, OF: 7.55 ± 1.96 mg.kg^−1^). Similar results were obtained for indoor-grown tomatoes. In this case, the data of two seasons, 2015 and 2016, were compared. Significantly lower concentrations of the two tocopherol derivatives were measured for most cultivars in 2016 compared to 2015 (in 2015—8.93 mg.kg^−1^ α-tocopherol; 1.74 mg.kg^−1^ γ-tocopherol and in 2016—6.41 mg.kg^−1^ α-tocopherol; 1.25 mg.kg^−1^ γ-tocopherol mean values, respectively) (Table 2).

Comparison of industrial and fresh market tomatoes showed that the α-tocopherol content of processing varieties is higher, but the difference is not significant at the 0.01 level. If we compare the varieties of different fruit sizes, our results show that the amount of γ-tocopherol is the highest in the small-fruited cherry varieties and the lowest in the large-fruited varieties in all three years examined. This variation is most likely due to the differences in the dry matter content of the fruits. It is of special interest that no significant differences in tocopherols were noticed between the resistant and susceptible varieties (Table 2).

### 2.3. Content of Vitamin C

Physiologically, vitamin C acts as a water-soluble antioxidant and is a cofactor of numerous enzymes. Evidence for in vivo antioxidant functions of ascorbate includes scavenging of reactive oxidants in activated leukocytes, lung and gastric mucosa, and reduced lipid peroxidation, as measured by urinary isoprostane excretion [37]. To provide antioxidant protection, the recommended dietary allowance (RDA) is 90 mg/day for adult men and 75 mg/day for adult women, while the tolerable upper intake level (UL) for adults is 2 g/day [37].

The amount of vitamin C is affected by the duration of storage, the temperature of transport, and possible injuries. According to the results of Ntagkas and coworkers [44], the ascorbic acid content of tomatoes increases under better light conditions with a higher number of hours of sunshine. Valšíková-Frey [45] had shown that the production of vitamin C is also affected by fruit maturity, seasons, soil, and agricultural engineering. Our results showed the same tendency: the amount of vitamin C in tomatoes is also influenced by genetic background, cultivation mode, and environmental parameters. Based on the data found in the literature, the vitamin C content of fresh tomato fruits varies between 60 and 500 mg.kg^−1^ [45,46,47,48]. The results we received correspond to this range; we measured a vitamin C content between 60.8 and 312 mg.kg^−1^, and the only outlier was the content of 700 mg.kg^−1^, measured in Cherrola K1 fruit in 2016. Cherrola K1 contained the most vitamin C (mean value 367.9 mg.kg^−1^), while the NAIK114 variety had the least (mean value 90.4 mg.kg^−1^) of the 14 tomato varieties examined in the studied seasons. Pandey et al. [49] studied the anthocyanin and vitamin C content in strawberries under different growing conditions. They found that fruits from open field conditions recorded higher total and reducing sugar and vitamin C content. However, our results did not prove the same; in 2016 we actually measured higher average concentration of vitamin C (PH: 225 mg.kg^−1^, OF: 265 mg.kg^−1^) in the open field samples for almost all varieties, but in 2015 we did not find a significant difference between the two cultivation methods (Table 3).

The impact of seasonal variations on the vitamin C concentration could be noticed in different years only in the outdoor cultivation. In 2016, we measured a significantly higher concentration of vitamin C (265 mg.kg^−1^ mean value) compared to other years (207 mg.kg^−1^ as average value in 2014, 134 mg.kg^−1^ as average value in 2015). In the case of indoor-grown tomato, there was not a significant difference for all varieties, which indicates a more balanced situation of environmental parameters (Table 3).

No significant difference was found between the vitamin C content of freshly consumable and industrial tomato varieties. However, the varieties of different sizes differed significantly in 2014. Small varieties contained the most vitamin C (274 mg.kg^−1^), while large varieties have the least (100.8 mg.kg^−1^). Such a trend was similar in the other two examined years, but the differences were not significant. Like with vitamin E, no significant differences were found between resistant and susceptible varieties.

### 2.4. Content of Polyphenols and Antioxidant Capacity

Reactive free radicals could be causative agents of many human diseases, including coronary heart disease and cancer. It has been suggested that the consumption of fruits and vegetables, the main sources of antioxidants in the diet, can reduce the potential stress caused by free radicals [50]. One of the main characteristics of antioxidant components in tomatoes, such as carotenoids, tocopherols, polyphenols, and vitamin C, is their antioxidant potential, either in lipophilic or hydrophilic parts [51]. From a methodological point of view, the stable DPPH radical-scavenger model is suitable for measuring the antioxidant activity of plants and foods [52,53,54].

Sanchez-Moreno and coworkers [55] found that the radical-scavenging capacity was higher in the aqueous fractions of tomatoes than in the fat-soluble fractions of tomato juice. Vitamin C was mainly responsible for the radical-scavenging ability of the water-soluble fraction of tomato juice, while carotenoids, lutein, and lycopene were also responsible for the kinetics of DPPH changes in the fat-soluble fractions of tomato juice. The accumulation of polyphenols and l-ascorbic acid was evaluated by Martí et al. [56] during traditional and organic farming, and it was established that the effect of the genotype was significantly greater than the cultivation system, and even decisive.

The antioxidant capacity and polyphenol content of the Cherrola F1 variety were exceptionally high (2.49 mM Tr/kg and 621 GAE/kg mean value), while the processing Unorosso (0.8 mM Tr/kg and 311.1 GAE/kg mean value) and NAIK114 fresh market line (1.23 mM Tr/kg and 282.7 GAE/kg mean value) varieties showed the lowest values (Table 4).

No significant differences were found in the total polyphenol values and the trend also changed by investigated variety. For some cultivars, such as Cherrola F1, the total polyphenol content was significantly higher in 2014 (804.12 ± 16.78; 606.24 ± 53.12; 579.19 ± 44.2 mg GAE/kg), while for other cultivars there was no significant difference between the three investigated years, such as Mokka F1 (395.71 ± 31.58; 339.36 ± 53.18; 348.14 ± 29.67 mg GAE/kg).

Neither the total polyphenols nor the antioxidant values were found to differ significantly between the tomato samples grown in the three investigated years. Although the mean antioxidant capacity and total polyphenol content of the field samples were higher than those of the plastic house samples, the difference was not significant for all varieties. Since the amount of phenolics increases in tomato under abiotic and biotic stress [57], the controlled conditions in the greenhouse explain the lower DPPH and total polyphenol values (Table 3). Slight differences could be found among the fresh market, cherry, and industrial varieties in any of the parameters studied.

The DPPH radical-scavenging activity of the small berry-sized varieties was measured in the range 0.97–4.4 mMTr/kg and of the large berry-sized varieties in the range 0.79–2.71 mMTr/kg in both farming technologies. Total polyphenol content ranged from 278 to 804 and from 240 to 461 mg GAE/kg, respectively (Table 3), in the same cultivar groups. The varieties Elan and NAIK114, which belong to the large berry-sized varieties, on the other hand, contain the smallest amount of the same components compared to the other varieties. Both the antioxidant capacity and the total polyphenol value are inversely proportional to the increase in the size of the tomatoes, but the difference between the groups is also not significant. However, it is interesting that both parameters were lower in the resistant varieties than in the susceptible varieties, which can also be explained by the effect of abiotic/biotic stress.

## 3. Discussion

**Figure 1:**
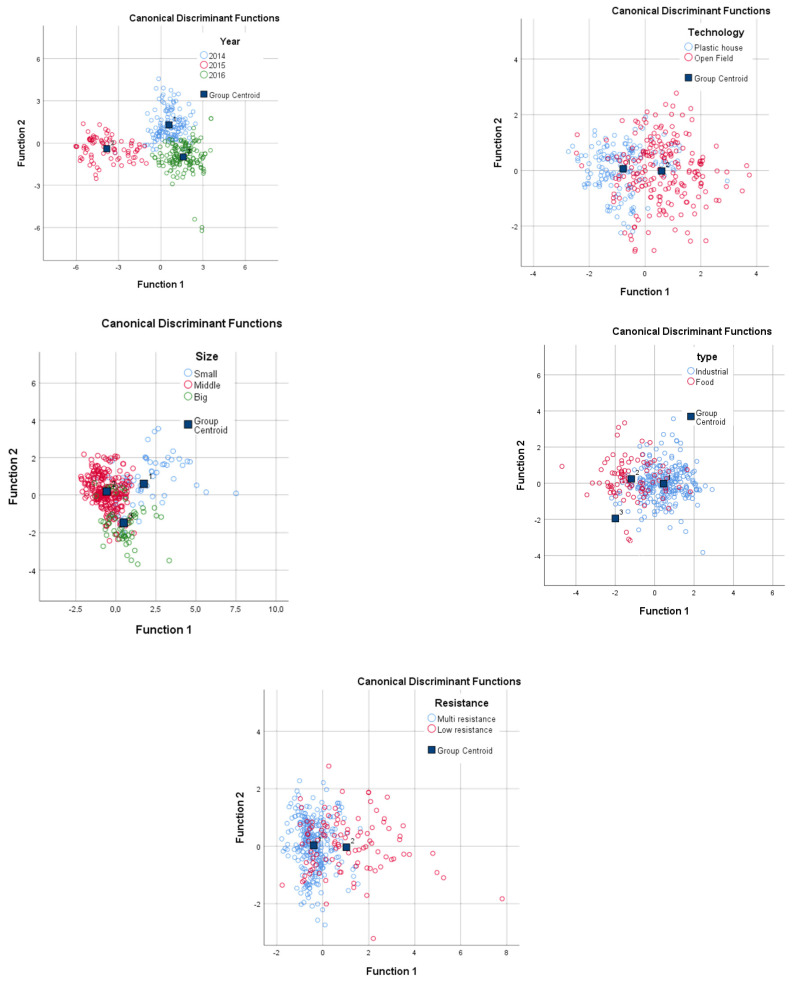
Discriminant analysis of different season, field technology, size, type, and resistance data of tomato samples. Discriminant analysis of the results indicated that 95.8% (Year); 78.1% (Technology), 77.2% (Fruit size), 77.7% (Type), 80.3% (Resistance) of original grouped cases were correctly classified.

Based on the 3-year summarized data of all measured parameters, the discriminant analysis was able to classify the season by 95.8% probability from the measured data (Figure 1).

It can be said that industrial varieties are richer in α-tocopherol and carotenoids (lycopene), except for the Cherrola F1 variety, which were proved by correlation analysis as well (Table 4). Based on the results of the discriminant analysis, the different types can be grouped with 80–94% probability, but the joint evaluation of the three seasons also groups the varieties with at least 77% probability.

Our results also prove that small-sized varieties such as, e.g., the values of Cherrola F1, NAIK 3254 F1, and Prairie Scooner cultivars’ β-carotene, γ-tocopherol, DPPH, vitamin C, and total polyphenol, were significantly higher than the other cultivars, in almost all seasons, for both cultivation methods (Table 4). For lycopene and α-tocopherol, no correlation was found between concentration and berry size. Summarizing the 3-year data, we can properly identify 74.7% of the samples based on the discriminant analysis based on all the measurements. However, if lycopene and α-carotene values were not considered in the calculations, this value was reduced to 60%. Examined separately for each year, we obtained a value above 90% for all three seasons.

Among the tomato genotypes examined were disease-susceptible or few resistance-carrying varieties (e.g., Cherrola, Elan, Prairie Schooner, NAI 3254, Unorosso) and those resistant to at least five fungal and bacterial diseases (e.g., NAIK 114, NAIK1122, Aragon F1, NAIK 3355). Comparing the average values of resistant and susceptible cultivars, we found that there is no significant difference in tocopherol concentration and antioxidant capacity between the two groups. The average values of polyphenols, β-carotene, and vitamin C are higher in susceptible varieties. Susceptible varieties include more small sizes than medium or large fruit-size varieties, which can cause higher b-carotene concentrations. Statistical evaluation of our results showed that in all three years, the varieties with resistant and susceptible traits can be distinguished with 80.3% probability. This result demonstrated that in resistant varieties, not only the components involved in protection system are synthesized, but they also affect other components of the tomato fruit.

Based on the discriminant analysis of the results, the various years, technology, and investigated factors could be distinguished by analysis of secondary metabolites. The discriminant analysis based on the 3-year data was able to distinguish the season by 95.8% probability from the measured data. Based on the discriminant analysis of the 3-year data, it was possible to differentiate the samples with a probability of about 80% according to the method of cultivation, the size of the fruit, and the method of use. By evaluating the results separately each year, the samples belonging to different groups can be distinguished with even greater certainty. It was a remarkable conclusion that the metabolite composition of the resistant varieties and breeding lines also differed from the susceptible varieties. The susceptible varieties contain higher concentrations of polyphenols than resistant tomato lines. Even when evaluating the aggregate 3-year sample set, the two cultivar groups can be distinguished with 80% confidence. During the evaluation of the tomato varieties, the small berry-sized and susceptible Cherrola F1 variety contained, on average, the highest amount of the investigated bioactive components. Among the newly bred resistant hybrids, the ZKI114 variety proved to be the most outstanding in the amounts of bioactive components.

## 4. Materials and Methods

### 4.1. Plant Material and Technology

Cultivation of tomato plants was carried out in the experimental field of the National Agricultural Research and Innovation Center Vegetable Crop Research Department, Kecskemét Station. The open field (outdoor) and plastic house (indoor) experiments were conducted in the loose sandy soil (KA: 31) of the experimental field during 2014, 2015, and 2016. During the summer months, green-colored rashel net cover was applied to reduce the inner temperature of the plastic house.

Professional hybrids, test hybrids, and parental lines representing fresh market (3), cherry (3), and processing (8) types had been used in the experiment (Table 5), carrying no, few, or multiple resistances against different diseases. No high-lycopene genotype was studied. Seedlings were grown in a heated greenhouse, for 5 weeks and P-dominant Ferticare growing solution had been applied. The seedlings were planted each year in mid-April and mid-May with 25 plants per genotype.

Fertilizers were given via drip irrigation according developmental phase following Yara (Yara UK Limited, UK) recommendations with the frequency 6–7 times per week (plastic house) and 2–3 times a week (open field). Daily water requirement of the crop was calculated by the Helyes and Varga [58] formula (average daily temperature in Celsius/5 = water expressed in mm). No ripening acceleration treatment was applied.

In mid-August, 25–25 randomly selected red ripe fruits were collected during each year for three replicates from the genotypes examined.

### 4.2. Chemicals

Standard ascorbic acid, lycopene, β-carotene, tocopherol standard solutions (of α, β, γ, δ), Folin–Ciocalteu reagent, gallic acid, DPPH (2,2-diphenyl-1-picrylhydrazyl), and Trolox (6-hydroxy-2,5,7,8-tetra-methylchroman-2-carboxylic acid) were purchased from Sigma-Aldrich Ltd. (St. Louis, MI, USA). HPLC and analytical-grade organic solvents (methanol, 1,2-dichloroethan, acetonitrile, hexane, ethanol, acetone) and other chemicals (quartz sand, KH_2_PO_4_, anhydrous Na_2_SO_4_, metaphosphoric acid—analytical grade, Na_2_CO_3_) were purchased from VWR International LLC (Radnor, PA, USA).

### 4.3. Analytical Methods

Sample preparation of tomato. The tomato fruits were collected at fully mature stage and transported immediately to the laboratory. The fruits were homogenized using a blender and the samples were stored at −20 °C until analysis. One test sample was obtained by homogenizing tomatoes from 4–5 different plants; the measurements were performed with 3 parallel test samples.

Measurement of polyphenol content. Total polyphenols were quantified using the Folin–Ciocalteu method [59]. Samples were weighed (2.0 g), and extracted with 80% (*v*/*v*) methanol (filled up to 20 mL volume) at 4 °C for 24 h. Afterward, 100 μL of the methanol extracts, 0.5 mL of Folin–Ciocalteu reagent, 2 mL of sodium carbonate solution (20% *w*/*v*), and distilled water were mixed up to 10 mL final volume. The tubes were left at room temperature for 60 min, and the absorbance of the samples was measured at 750 nm. Polyphenol content was given in gallic acid equivalent (GAE)/per 1000 g sample.

DPPH radical-scavenging activity. Methanol extracts (80% *v*/*v*) were used to assess the antioxidant capacity by the DPPH radical-scavenging method [60] with some modifications. A 50 μL aliquot of the extract and 2 mL of DPPH (0.1 mM) were shaken at 37 °C for 30 min and the absorbance was measured at 517 nm using a Jasco Spectrophotometer (Model 7850). The calibration curve was prepared with 80% (*v*/*v*) methanol solution of Trolox (6-hydroxy-2,5,7,8-tetra-methylchroman-2-carboxylic acid) at 100–1000 μmol/l concentrations. The antioxidant capacity was expressed as mmol Trolox equivalents (TE)/1000 g sample fw.

Ascorbic acid determination. Ascorbic acid content was determined using the RP-HPLC method with UV detection described by Nagy et al. [61]. Five grams of well-homogenized tomato sample (5 fruits per replication) was ground in a crucible mortar with 1 g quartz sand. The mixture was poured into 50 mL metaphosphoric acid (3% *w*/*v*, analytical grade, temperature 4 °C) and transferred to a 100 mL Erlenmeyer flask. Samples were shaken (15 min) and then filtered. In addition, the filtrate was purified with a 0.45 mm PTFE syringe filter before injection into an HPLC column. The analytical determination of ascorbic acid was performed using a C18 Nautilus 100–5 μm column (150 mm × 4.6 mm i.d.) (Macherey-Nagel, Duren, Germany) with gradient elution of 0.01 M KH_2_PO_4_ (A) and acetonitrile (B). The gradient elution started with 1% B in A and changed to 30% B in A after 15 min; it then turned to 1% A in B after 5 min. The flow rate was 0.7 mL/min. The absorption maximum of ascorbic acid under these conditions was detected at 265 nm. For quantitative determination of ascorbic acid internal standard calibration was used.

Determination of carotenoids. Separation and analysis of carotenoids was performed according to Daood and coworkers [62] using an EC 150/4.6 Nucleodur C18 Isis 3 μm column (Macherey Nagel, Düren, Germany) and gradient elution (A: methanol; B: water, C: isopropanol–acetone–methanol) RP-HPLC method with UV detection. Peak identification was based on comparison of retention time and spectral characteristics of available standards (lycopene, β-carotene), which were also used for the quantification of lycopene and β-carotene. Five grams of well-homogenized (5 fruits per replications) tomato fruit were crushed in a crucible mortar in the presence of 1 g quartz sand and 0.5 g ascorbic acid. The extraction procedure started with binding water with methanol followed by extraction of carotenoids by 1,2-dichloroethane in a liquid–liquid extraction. The polar and non-polar phases were separated by addition of 1 mL of distilled water and mechanical shaking for 15 min. The lower non-polar phase was separated in a separating funnel, dried through anhydrous Na_2_SO_4_, and evaporated at 30 °C under vacuum. The residue was redissolved in HPLC grade acetone and filtered through a 0.45 μm Teflon syringe filter before injection. Internal standard calibrations were used for quantitative determination of carotenoids.

Determination of tocopherols. To analyze tocopherols, the tomato lipid fraction obtained by the same procedure used for carotenoid extraction was saponified with KOH and extracted by n-hexane and analyzed using the NP-HPLC method with an EC 250/4.6 Nucleosyl 100–5 column (Macherey Nagel, Germany) and isocratic elution (n-hexane: ethanol—99.6:0.4) and fluorescent detection (Ext: 295 nm, Em: 320 nm) according to the procedure described by Abushita et al. [63]. Two grams of tomato samples were saponified with a mixture of 5 mL of 30% methanolic KOH solution, 20 mL of methanol, and 0.5 g of ascorbic acid for 25 min from boiling. Fifteen milliliters of 20% NaCl solution was added to the mixture and then extracted with 2 × 40 mL of hexane; the organic phase was evaporated to dryness. The residue was redissolved in HPLC-grade hexane and filtered through a 0.45 μm Teflon syringe filter before injection. Internal standards were used for quantitative determination of tocopherols.

A Waters Alliance liquid chromatographic instrument consisting of a Model 2696 Separation Module (gradient pump, auto-sampler, and column heater) and a Model 2695 photodiode-array detector was used for the analysis of carotenoids and vitamin C. Operation and data processing were performed by Empower software. For tocopherol analysis, a combination of a Beckman 114 M isocratic pump, a model RF-535 Shimadzu fluorometric detector, and a Waters-740 Data Module integrator was used.

Statistical analysis. For spectrophotometric and HPLC analysis, all the samples were analyzed in triplicates. All data in the tables were presented as a mean number (n = 3) and standard deviations. IBM SPSS Statistics (USA, SPSS) software was used for normality analysis (Kolmogorov–Smirnov and Shapiro–Wilk test), Pearson’s correlation analysis, ANOVA (Tukey test), t-probe, and discriminant analysis.

## Figures and Tables

**Table 1:** Lycopene and β-carotene concentrations in tomato samples during 2014–2016 under different growing technology (NS—no samples; OF—open field; PH—plastic house).

Genotype	Growing Technology	2014		2015		2016	
		Lycopene (mg.kg^−1^)	β-Carotene (mg.kg^−1^)	Lycopene (mg.kg^−1^)	β-Carotene (mg.kg^−1^)	Lycopene (mg.kg^−1^)	β-Carotene (mg.kg^−1^)
NAIK 3992	OF	31.12 ± 0.61	1.05 ± 0.04	42.76 ± 3.2	2.95 ± 0.22	50.84 ± 4.04	1.93 ± 0.25
	PH	NS	NS	48.05 ± 0.51	2.80 ± 0.10	49.95 ± 1.18	2.42 ± 0.12
NAIK 114	OF	9.41 ± 0.08	0.83 ± 0.04	26.14 ± 8.51	2.81 ± 0.06	10.38 ± 0.95	1.54 ± 0.36
	PH	NS	NS	15.83 ± 0.89	1.53 ± 0.25	NS	NS
NAIK 1122	OF	11.29 ± 0.64	0.84 ± 0.07	42.51 ± 3.13	3.02 ± 0.92	31.91 ± 5.26	2.05 ± 0.63
	PH	NS	NS	39.25 ± 1.29	3.12 ± 0.42	23.86 ± 0.63	1.41 ± 0.01
NAIK 352	OF	63.23 ± 5.71	1.79 ± 0.13	20.76 ± 6.46	3.82 ± 1.12	48.21 ± 0.55	1.74 ± 0.08
	PH	7.46 ± 0.25	0.43 ± 0.02	43.05 ± 4.95	1.90 ± 0.32	53.82 ± 3.01	2.92 ± 0.14
Aragon F1	OF	31.2 ± 0.40	1.43 ± 0.03	46.38 ± 0.67	6.25 ± 0.97	33.49 ± 2.61	3.67 ± 0.52
	PH	NS	NS	42.56 ± 2.46	3.52 ± 1.04	43.13 ± 0.32	2.78 ± 0.14
NAIK 3355	OF	35.79 ± 0.83	1.12 ± 0.04	47.10 ± 1.88	4.18 ± 0.23	51.30 ± 0.48	3.05 ± 0.09
	PH	NS	NS	39.90 ± 3.46	3.30 ± 0.51	32.40 ± 1.33	2.45 ± 0.23
Cherrola F1	OF	16.12 ± 0.64	1.54 ± 0.01	31.04 ± 9.45	11.66 ± 3.34	16.67 ± 2.57	3.39 ± 0.54
	PH	NS	NS	36.29 ± 1.83	6.56 ± 0.13	26.30 ± 0.85	4.43 ± 0.07
Elán F1	OF	NS	NS	28.15 ± 2.66	4.92 ± 0.71	25.30 ± 2.13	4.43 ± 0.04
	PH	15.48 ± 0.11	1.39 ± 0.07	43.86 ± 1.34	4.12 ± 0.5	26.98 ± 0.64	2.79 ± 0.08
Prairie	OF	11.23 ± 0.38	0.58 ± 0.02	49.01 ± 7.5	6.47 ± 0.53	29.85 ± 14.57	3.89 ± 0.92
Schooner	PH	NS	NS	43.74 ± 3.28	5.43 ± 0.67	32.02 ± 15.78	2.18 ± 0.65
NAIK 3254	OF	31.88 ± 2.18	1.67 ± 0.13	47.12 ± 4.53	6.05 ± 1.49	43.87 ± 4.25	5.40 ± 0.93
	PH	NS	NS	38.49 ± 0.75	5.30 ± 0.93	49.10 ± 4.22	6.44 ± 1.68
Unorosso F1	OF	NS	NS	42.71 ± 4.87	4.38 ± 0.58	38.22 ± 1.23	2.43 ± 0.29
	PH	43.60 ± 3.01	0.69 ± 0.03	40.20 ± 2.23	2.59 ± 0.29	NS	NS
NAIK 3451	OF	8.05 ± 0.16	0.49 ± 0.02	39.67 ± 6.51	3.43 ± 0.35	45.94 ± 2.01	3.91 ± 0.24
	PH	NS	NS	46.58 ± 1.86	1.60 ± 0.23	48.38 ± 5.27	2.48 ± 0.20
NAIK 3270	OF	12.70 ± 0.31	0.51 ± 0.02	44.62 ± 5.39	3.11 ± 0.27	40.91 ± 1.69	1.49 ± 0.19
	PH	NS	NS	46.94 ± 2.22	2.61 ± 0.8	45.17 ± 6.92	1.91 ± 0.55
Mokka F1	OF	19.69 ± 0.59	0.89 ± 0.05	28.37 ± 10.41	1.91 ± 1.03	35.01 ± 2.31	3.05 ± 0.31
	PH	NS	NS	39.95 ± 0.89	2.49 ± 0.00	30.18 ± 0.97	0.65 ± 0.02

**Table 2:** The α- and γ-tocopherol concentration in tomato samples during 2014–2016 under different growing technology (NS—no samples; OF—open field; PH—plastic house).

Genotype	Growing Technology	2014		2015		2016	
		α-Tocopherol (mg.kg^−1^)	γ-Tocopherol (mg.kg^−1^)	α-Tocopherol (mg.kg^−1^)	γ-Tocopherol (mg.kg^−1^)	α-Tocopherol (mg.kg^−1^)	γ-Tocopherol (mg.kg^−1^)
NAIK 3992	OF	18.99 ± 0.23	7.61 ± 0.09	8.09 ± 0.36	0.31 ± 0.03	9.34 ± 0.47	1.09 ± 0.06
	PH	NS	NS	9.27 ± 0.29	1.74 ± 0.04	4.62 ± 0.12	0.95 ± 0.06
NAIK 114	OF	9.02 ± 0.18	1.72 ± 0.05	6.71 ± 0.15	0.41 ± 0.01	6.93 ± 0.28	0.64 ± 0.05
	PH	NS	NS	6.14 ± 0.23	1.41 ± 0.03	NS	NS
NAIK 1122	OF	8.10 ± 0.03	3.01 ± 0.04	11.30 ± 0.16	0.80 ± 0.03	5.25 ± 0.41	0.46 ± 0.02
	PH	NS	NS	9.96 ± 0.26	0.93 ± 0.04	4.38 ± 0.21	1.62 ± 0.03
NAIK 352	OF	18.10 ± 0.41	7.28 ± 0.05	8.13 ± 0.12	0.88 ± 0.02	8.17 ± 0.48	0.63 ± 0.03
	PH	11.50 ± 0.35	5.12 ± 0.43	7.54 ± 0.35	1.98 ± 0.14	5.29 ± 0.03	2.60 ± 0.03
Aragon F1	OF	18.44 ± 0.52	3.86 ± 0.1	12.15 ± 0.33	1.52 ± 0.07	11.77 ± 0.64	0.88 ± 0.03
	PH	NS	NS	8.63 ± 0.24	0.72 ± 0.02	6.66 ± 0.41	0.77 ± 0.08
NAIK 3355	OF	10.98 ± 0.55	2.61 ± 0.16	12.00 ± 0.12	0.84 ± 0.03	8.13 ± 0.62	0.44 ± 0.01
	PH	NS	NS	5.09 ± 0.12	1.02 ± 0.03	9.21 ± 0.16	0.28 ± 0.01
Cherrola F1	OF	15.31 ± 0.25	8.50 ± 0.08	9.16 ± 0.18	3.89 ± 0.06	6.82 ± 0.06	2.28 ± 0.07
	PH	NS	NS	7.78 ± 0.28	4.74 ± 0.14	4.03 ± 0.20	3.05 ± 0.16
Elán F1	OF	NS	NS	8.50 ± 0.18	2.16 ± 0.06	3.30 ± 0.18	1.55 ± 0.10
	PH	6.99 ± 0.27	2.53 ± 0.09	6.52 ± 0.12	1.29 ± 0.04	2.56 ± 0.09	0.86 ± 0.05
Prairie	OF	14.81 ± 0.12	1.63 ± 0.06	11.35 ± 0.22	2.59 ± 0.03	6.95 ± 0.23	1.30 ± 0.03
Schooner	PH	NS	NS	11.69 ± 0.27	0.70 ± 0.06	5.96 ± 0.38	1.46 ± 0.11
NAIK 3254	OF	13.95 ± 0.35	9.35 ± 0.05	12.42 ± 0.20	2.58 ± 0.04	7.03 ± 0.50	1.72 ± 0.10
	PH	NS	NS	8.70 ± 0.24	1.19 ± 0.05	5.85 ± 0.45	0.81 ± 0.08
Unorosso F1	OF	NS	NS	7.64 ± 0.19	1.26 ± 0.03	8.09 ± 0.57	1.27 ± 0.12
	PH	6.65 ± 0.36	2.49 ± 0.14	9.43 ± 0.03	3.52 ± 0.01	NS	NS
NAIK 3451	OF	15.24 ± 0.26	5.60 ± 0.07	10.80 ± 0.17	0.54 ± 0.03	6.97 ± 0.26	0.65 ± 0.03
	PH	NS	NS	7.86 ± 0.28	0.95 ± 0.03	3.86 ± 0.23	1.65 ± 0.15
NAIK 3270	OF	18.69 ± 0.19	5.04 ± 0.03	8.13 ± 0.31	4.68 ± 0.12	6.93 ± 0.42	2.38 ± 0.15
	PH	NS	NS	9.16 ± 0.29	2.76 ± 0.06	5.29 ± 0.20	1.00 ± 0.08
Mokka F1	OF	12.22 ± 0.18	3.61 ± 0.01	7.55 ± 0.18	1.67 ± 0.05	10.05 ± 0.69	1.06 ± 0.07
	PH	NS	NS	8.38 ± 0.15	1.71 ± 0.01	3.35 ± 0.17	1.16 ± 0.05

**Table 3:** Antioxidant capacity (DPPH), polyphenol, and vitamin C concentrations in tomato samples during 2014–2016 under different growing technology (NS—no samples; OF—open field; PH—plastic house).

Genotype	Growing Technology	2014			2015			2016		
		DPPH (mMTr/kg)	Polyphenol (mg GAE/kg)	Vitamin C (mg.kg^−1^)	DPPH (mMTr/kg)	Polyphenol (mg GAE/kg)	Vitamin C (mg.kg^−1^)	DPPH (mMTr/kg)	Polyphenol (mg GAE/kg)	Vitamin C (mg.kg^−1^)
NAIK 3992	OF	4.36 ± 0.04	496.32 ± 13.33	219.02 ± 11.83	0.97 ± 0.17	371.67 ± 14.19	142.98 ± 6.5	1.92 ± 0.03	404.32 ± 31.9	224.69 ± 11.14
	PH	NS	NS	NS	0.76 ± 0.21	287.58 ± 36.56	99.59 ± 21.37	1.10 ± 0.28	276.32 ± 38	215.19 ± 6.17
NAIK 114	OF	2.71 ± 0.57	320.1 ± 11.39	72.39 ± 31.69	1.04 ± 0.3	310.33 ± 3.68	81.72 ± 15.21	1.24 ± 0.38	348.98 ± 18.9	186.60 ± 15.53
	PH	NS	NS	NS	0.79 ± 0.16	239.00 ± 33.27	67.18 ± 12.78	NS	NS	NS
NAIK 1122	OF	2.11 ± 0.35	449.62 ± 15.96	141.83 ± 35.28	1.59 ± 0.29	369.08 ± 13.82	175.48 ± 16.73	2.06 ± 0.38	461.02 ± 18.6	330.80 ± 34.27
	PH	NS	NS	NS	2.38 ± 0.16	396.03 ± 52.17	179.21 ± 14.63	0.79 ± 0.03	236.84 ± 16.3	148.95 ± 6.65
NAIK 352	OF	0.56 ± 0.03	428.1 ± 13.98	309.3 ± 9.93	1.11 ± 0.3	346.82 ± 37.62	60.81 ± 17.87	1.87 ± 0.04	345.46 ± 37.1	249.67 ± 24.01
	PH	0.47 ± 0.06	361.39 ± 39.74	58.08 ± 11.7	0.78 ± 0.15	245.25 ± 10.84	94.20 ± 8.11	0.52 ± 0.04	232.03 ± 20.8	170.70 ± 9.24
Aragon F1	OF	3.02 ± 0.30	394.58 ± 109.08	186.01 ± 4.25	1.56 ± 0.27	391.03 ± 26.67	171.72 ± 21.33	2.22 ± 0.28	416.85 ± 7.03	336.25 ± 21.01
	PH	NS	NS	NS	2.34 ± 0.06	405.90 ± 53.06	137.86 ± 26.97	1.16 ± 0.16	282.93 ± 34.1	198.01 ± 30.86
NAIK 3355	OF	1.73 ± 0.30	407.09 ± 31.79	236.51 ± 10.27	1.02 ± 0.22	459.61 ± 68.03	135.11 ± 20.45	1.86 ± 0.15	314.08 ± 36.5	354.28 ± 12.51
	PH	NS	NS	NS	0.53 ± 0.09	371.61 ± 29.18	96.59 ± 7.02	3.41 ± 0.22	377.23 ± 20.9	244.65 ± 8.61
Cherrola F1	OF	1.99 ± 0.35	804.12 ± 16.78	289.85 ± 17.2	2.48 ± 0.19	606.24 ± 53.12	204.38 ± 28.64	3.97 ± 0.2	579.19 ± 44.2	191.08 ± 10.52
	PH	NS	NS	NS	2.3 ± 0.13	611.32 ± 35.5	312.25 ± 19.64	2.01 ± 0.04	546.30 ± 64	702.61 ± 22.39
Elán F1	OF	NS	NS	NS	1.13 ± 0.23	354.46 ± 24.22	175.74 ± 16.24	1.28 ± 0.31	402.81 ± 76.5	176.66 ± 8.07
	PH	0.33 ± 0.04	363.24 ± 13.68	88.34 ± 3.45	1.06 ± 0.16	401.98 ± 31.41	165.25 ± 15.89	0.84 ± 0.15	336.15 ± 24.5	223.11 ± 5.47
Prairie	OF	2.71 ± 0.38	565.2 ± 63.33	226.21 ± 9.83	1.39 ± 0.1	377.35 ± 52.1	86.79 ± 15.06	2.07 ± 0.16	324.57 ± 8.04	235.53 ± 19.36
Schooner	PH	NS	NS	NS	0.97 ± 0.21	414.01 ± 16.85	161.75 ± 29.11	1.52 ± 0.17	293.64 ± 49.5	141.69 ± 13.19
NAIK 3254	OF	4.40 ± 0.47	769.06 ± 74.55	306.44 ± 56.87	1.65 ± 0.4	449.21 ± 67.83	156.87 ± 13.97	2.16 ± 0.18	423.67 ± 25.95	371.30 ± 20.19
	PH	NS	NS	NS	2.11 ± 0.35	365.59 ± 20.77	293.04 ± 7.5	1.27 ± 0.16	278.04 ± 10.48	176.25 ± 5.51
Unorosso F1	OF	NS	NS	NS	0.63 ± 0.05	340.22 ± 59.45	131.15 ± 14.57	1.41 ± 0.22	250.63 ± 44.08	136.86 ± 20.85
	PH	0.51 ± 0.05	295.33 ± 16.36	64.49 ± 12.91	0.66 ± 0.14	358.35 ± 35.56	144.80 ± 22.45	NS	NS	NS
NAIK 3451	OF	3.16 ± 0.83	429.73 ± 36.22	183.55 ± 25.08	1.34 ± 0.29	349.95 ± 26.28	138.12 ± 17.3	2.02 ± 0.15	338.19 ± 18.74	363.11 ± 18.98
	PH	NS	NS	NS	0.81 ± 0.22	339.72 ± 36.63	130.11 ± 15.63	0.91 ± 0.1	222.48 ± 23.47	185.41 ± 2.27
NAIK 3270	OF	3.82 ± 0.14	398.42 ± 53.33	137.61 ± 10.78	0.83 ± 0.09	290.11 ± 27.85	96.72 ± 12.81	1.84 ± 0.07	336.13 ± 12.92	210.39 ± 17.83
	PH	NS	NS	NS	0.75±	241.88 ± 32.23	76.80 ± 19.08	0.91 ± 0.03	303.47 ± 11.7	152.46 ± 0.36
Mokka F1	OF	3.02 ± 0.30	395.71 ± 1.89	177.97±	1.42±	339.36 ± 53.18	130.47 ± 26.11	2.23 ± 0.27	348.14 ± 29.67	347.15 ± 2.19
	PH	NS	NS	NS	0.53±	330.40 ± 58.62	153.80 ± 19.17	0.79 ± 0.11	352.72 ± 32.9	150.77 ± 4.6

**Table 4:** Pearson correlation analysis among bioactive components, resistance level, fielding technology fruit size, and type of tomato. ** Correlation is significant at the 0.01 level (2-tailed). * Correlation is significant at the 0.05 level (2-tailed).

Correlations 2014–2016											
	A-Tocopherol	G-Tocopherol	DPPH	Total Polyphenol	Lycopene	B-Carotene	Vitamin C	Resistance	Technology	Size	Type
a-tocopherol	1										
g-tocopherol	0.580 **	1									
DPPH	0.455 **	0.339 **	1								
polyfenol	0.469 **	0.557 **	0.541 **	1							
lycopene	−0.349 **	−0.457 **	−0.362 **	−0.355 **	1						
b-carotene	−0.113	−0.161 *	−0.040	0.109	0.477 **	1					
vitamin C	−0.035	0.098	0.330 **	0.365 **	0.056	0.158 *	1				
resistance	0.026	0.157	0.083	0.302 **	0.032	0.422 **	0.162 *	1			
technology	0.397 **	0.116	0.479 **	0.383 **	−0.259 **	0.019	0.157	−0.006	1		
size	−0.101	−0.209 *	−0.184 *	−0.319 **	−0.146	−0.314 **	−0.365 **	−0.591 **	0.047	1	
type	−0.200 *	−0.058	0.053	0.175 *	−0.360 **	0.104	0.030	0.197 *	0.069	0.121	1

**Table 5:** Basic information on the analyzed tomato samples. Resistance against: V = Verticillium dahlia, F0 = Fusarium oxysporum f.sp. Lycopersici race 0; F1 = Fusarium oxysporum f.sp. Lycopersici race 1; Pt = Pseudomonas syringae pv. Tomato; Tm = ToMV, TMV, N = Meloidogyne sp.; C5 = Fulvia fulva (=Cladosporium fulvum).

Genotype	Type	Size of Fruit (g)	Resistance
Elán F1	fresh market	90–100	-
Cherrola F1	fresh market	15–20	V, F_0_
NAIK parent line/NAIK 114	fresh market	120–140	V, F_0_, F_1_, Tm, C_5_, N
NAIK 1122	fresh market	110–130	V, F_0_, F_1_, Tm, C_5_, N
Prairie Schooner (gene bank)	fresh market	20–23	V, F_0_
NAIK 3254	industrial	20–22	V, F_0_
Aragon F1	industrial	70–80	V, F_0_, F_1_, Pt, N
NAIK parent line/NAIK 352	industrial	60–65	V, F_0_, F_1_, Pt, N
Unorosso F1	industrial	60–70	V, F_0_
NAIK 3355	industrial	70–80	V, F_0_, F_1_, Pt, N
NAIK 3451	industrial	70–80	V, F_0_, F_1_, Pt, N
NAIK 3270	industrial	70–80	V, F_0_, F_1_, Pt, N
NAIK 3992	industrial	60–65	V, F_0_, F_1_, Pt, N
Mokka F1	industrial	75–80	V, F_0_, F_1_, Pt, N

## Data Availability

Data is contained within the article.
